# Being actively engaged in life in old age: determinants, temporal trends, and prognostic value

**DOI:** 10.1007/s40520-023-02440-9

**Published:** 2023-05-24

**Authors:** Ulla L. Aalto, Mia Knuutila, Tuuli Lehti, Anu Jansson, Hannu Kautiainen, Hanna Öhman, Timo Strandberg, Kaisu H. Pitkälä

**Affiliations:** 1 Department of Geriatrics, Helsinki University Hospital, Helsinki, Finland; 2 Department of General Practice and Primary Health Care, University of Helsinki, Helsinki, Finland; 3 Primary Health Care Unit, Helsinki University Hospital, Helsinki, Finland; 4 Social Services and Health Care, City of Helsinki, Helsinki, Finland; 5 Oulunkylä Rehabilitation Center, Helsinki, Finland; 6 The Finnish Association for the Welfare of Older Adults, Helsinki, Finland; 7 Center for Life Course Health Research, University of Oulu, Oulu, Finland

**Keywords:** Active engagement, Active ageing, Aged, Survival, Mortality

## Abstract

**Purpose:**

Recently, the concept of successful ageing has shifted from healthy ageing to active ageing, the latter emphasising even more the subjective perspective. Active agency is a marker for better functioning. However, the concept of active ageing lacks a clear definition so far. The specific aims of the study were to identify the determinants of being actively engaged in life (BAEL), to explore the changes in BAEL over 3 decades, and to explore the prognostic value of BAEL.

**Methods:**

This is a repeated cross-sectional cohort study of older (≥ 75 years) community-dwelling people in Helsinki in 1989 (*N* = 552), 1999 (*N* = 2396), 2009 (*N* = 1492), and 2019 (*N* = 1614). The data were gathered by a postal questionnaire at each time point. Being actively engaged in life was defined by two questions “Do you feel needed?” and “Do you have plans for the future?”, which was further converted into BAEL score.

**Results:**

An increasing temporal trend in BAEL score was observed through the study years. Male sex, good physical functioning and subjective health, and meaningful social contacts were determinants for higher BAEL score. Active agency measured by BAEL score predicted lower 15-year mortality.

**Conclusions:**

Older home-dwelling, urban Finnish people have become more actively engaged in recent years. The underlying causes are diverse but improved socioeconomic status observed over the study years was one of them. Social contacts and not feeling lonely were found to be determinants for being actively engaged. Two simple questions describing active engagement in life may help to predict mortality among older people.

## Introduction

The concept of successful and active ageing has been described as an individual being healthy and independent, free of disabilities and disease burden, and feeling satisfied with one’s life [1, 2]. In recent years, the discourse has shifted from the concept of successful ageing towards a broader concept of active ageing and being actively engaged in life [1, 3, 4]. There are several theories underlying the concept of active ageing; it can be approached either by research-derived clinical health measures and models, or more subjectively, the latter emphasising the individual’s own perceptions and values [5–8]. Multidimensional active ageing approaches include participation in several domains of life such as economically productive, social, physical, and leisure activities [9].

Social participation is considered one of the key indicators of active ageing determined by the WHO [1]. It is based on the assumption that older people also aim at being active participants in society, instead of being merely passive spectators. Maintaining a socially active lifestyle and participating in meaningful activities are ways to contribute to society. Social participation, understood as having social connections, participating in social activities, and volunteering, has also been associated with positive health outcomes such as higher subjective well-being and higher physical activity [10–12]. An important concept relating to social participation is self-efficacy, as it can be considered a means to accomplish an active life. Self-efficacy is defined as confidence in individual performance in certain domains. A person’s beliefs in one’s capacity and mastering one’s own life can help in adapting to and coping with events through the life-course. High self-efficacy is also considered to encourage and enable health-promoting behaviour in older adults [13]. Low self-efficacy, in turn, has been associated with greater risk of incident frailty or increased loneliness [14, 15]. In addition to differences in related theories, the concept of active ageing is also culture-dependent, which partly explains the lack of a unanimous definition and internationally validated scales [9, 16].

Since being actively engaged in life (BAEL) and mastering one’s own life are important markers for successful ageing, we aimed at exploring these concepts. We assume that these concepts contribute to healthy ageing and include dimensions such as self-efficacy and having active agency in one’s own life. Therefore, BAEL—not having a definite, pre-existing definition—was conceptualised by two subjective questions concerning feeling needed and having plans for the future. Specific aims of this study were (1) to explore the changes in BAEL in four cohorts over 3 decades, (2) to identify the determinants of BAEL in all cohorts, and (3) to explore the prognostic value of BAEL for mortality in the three cohorts (1989, 1999, and 2009).

## Methods

The current study is part of the Helsinki Ageing Study, which is a repeated cross-sectional cohort study started in 1989. It explores various determinants for health and successful ageing among older urban Finnish home-dwelling people in four cross-sectional cohorts from 1989 to 2019. The study population was recruited from the Finnish National Population Registry consisting of random samples of home-dwelling individuals aged 75 years or more living in Helsinki, Finland in 1989 (*n* = 552), 1999 (*n* = 2396), 2009 (*n* = 1492), and 2019 (*n* = 1614). In the first cohort (1989), the age groups 75, 80, and 85 years were included, whereas in the three latter cohorts (1999, 2009, and 2019) also, the age groups 90 and 95 years were included. The number of participants by age groups were 1681 (75 years), 1597 (80 years), 1422 (85 years), and 1354 (90 and 95 years). For data collection, postal surveys with identical questions were sent to eligible participants at each time point. The response rates for the postal surveys were 84% (1989), 80% (1999), 73% (2009), and 74% (2019). The response rates are based on an approximation of how many survey recipients had died, moved to another area, or moved to a nursing home between the latest population census and sample retrieval.

The study aimed at comparing four cross-sectional cohorts with respect to participants’ functioning, well-being, comorbidities, and attitudes towards life. The questionnaire gathered data on sociodemographics, e.g. education and marital status. Charlson Comorbidity Index (CCI) was calculated based on self-reported diagnoses, and it was used to indicate the burden of diseases [17]. The participants evaluated their self-rated health (SRH) on a four-point scale (healthy/quite healthy/quite unhealthy/unhealthy), which was further converted to either good (healthy/quite healthy) or poor (quite unhealthy/unhealthy) SRH.

Functional abilities were assessed by asking how participants were able to walk outdoors (easily/with difficulties either with devices or with another person’s help/not at all), and whether they needed other person’s daily help (yes/no). Psychosocial and social determinants, such as meeting with friends and loneliness, were also inquired. Being actively engaged in life (BAEL) was inquired by asking the following two questions: “Do you feel needed?” (yes/no) and “Do you have plans for the future?” (yes/no). BAEL score (0–2) was created by summing the number of “yes” responses.

Mortality data were retrieved from the central registers.

The study protocol was approved by the Helsinki University Hospital Ethics Committee.

### Statistics

Descriptive statistics are presented as means with SDs or as counts with percentages. Linearity across the three BAEL levels was evaluated using the Cochran–Armitage test (Chi-square test for trend), logistic models, and analysis of variance with an appropriate contrast (orthogonal). To determine characteristics associated with BAEL levels, multivariate ordered logistic regression models were applied. The Kaplan–Meier method was used to estimate the probability of survival in different BAEL categories. Cox proportional hazards regression was used to estimate the adjusted hazard ratios (HRs) and their 95% confidence intervals (CIs). Stata 17.0, StataCorp LP (College Station, TX, USA) statistical package was used for the analyses.

## Results

An increasing trend emerged in the proportion of older people having maximum BAEL score over 3 decades, as 27% in 1989, 40% in 1999, 38% in 2009, and finally 43% in 2019 gained 2 points in the BAEL score (*p* for linearity < 0.001, adjusted for age and sex) (Table 1).

**Table 1:** BAEL score in cohorts (1989, 1999, 2009, 2019) of the Helsinki Ageing Study

	1989, *N* = 552	1999, *N* = 2396	2009, *N* = 1492	2019, *N* = 1614
BAEL 0, *n* (%)	175 (32)	586 (24)	381 (26)	341 (21)
BAEL 1, *n* (%)	226 (41)	849 (35)	538 (36)	574 (36)
BAEL 2, *n* (%)	151 (27)	961 (40)	573 (38)	699 (43)

*p* for linearity < 0.001, adjusted for age and sex

*BAEL* being actively engaged in life

We combined all cohorts when exploring the characteristics and determinants associated with BAEL score. Females comprised 69% of all participants. Those with higher BAEL score, indicating being actively engaged in life, differed significantly from those with lower BAEL score in most demographic and health variables as well as in experienced loneliness. Those being actively engaged were more often male, younger, less often widowed, and had a higher level of education. They also reported less diagnosed diseases, thus having a lower CCI [17]. Better functioning in terms of cognitive function, ability to walk easily outdoors and being less dependent on other person’s daily help, and good SRH were also associated with a higher BAEL score. Practically none of those with BAEL score 2 suffered from loneliness, and a greater proportion of these individuals were active in meeting with friends compared with those with a lower BAEL score (Table 2).

**Table 2:** Characteristics of participants according to BAEL (being actively engaged in life) score (0–2)

	BAEL 0 (*N* = 1483)	BAEL 1 (*N* = 2187)	BAEL 2 (*N* = 2384)	*p*-value
Female, *n* (%)	1142 (77)	1525 (70)	1488 (62)	< 0.001
Age groups (years), *n* (%)				< 0.001
75	244 (16)	512 (23)	925 (39)	
80	295 (20)	588 (27)	714 (30)	
85	412 (28)	577 (26)	433 (18)	
90 and 95	532 (36)	510 (23)	312 (13)	
Widowed, *n* (%)	816 (56)	918 (43)	777 (33)	< 0.001
Education < 8 years, *n* (%)	703 (48)	1044 (49)	822 (35)	< 0.001
Dementia diagnosis, *n* (%)	357 (24)	345 (16)	224 (9)	< 0.001
CCI^a^, mean (SD)	2.3 (1.9)	1.9 (1.8)	1.6 (1.7)	< 0.001
Able to easily walk outdoors, *n* (%)	563 (39)	1330 (62)	1836 (78)	< 0.001
Needs daily help, *n* (%)	448 (31)	397 (19)	245 (10)	< 0.001
Self-rated health good, *n* (%)	817 (58)	1650 (78)	2038 (88)	< 0.001
Suffering from loneliness constantly, *n* (%)	189 (13)	65 (3)	11 (0)	< 0.001
Meeting regularly with friends, *n* (%)	681 (47)	1359 (64)	1794 (77)	< 0.001

^a^Charlson comorbidity index [17]

Table 3 shows the determinants of BAEL score in the multivariate ordered logistic regression model. In the whole population, older age, being widowed, low level of education, and loneliness decreased the odds of being actively engaged in life. Male sex, good physical functioning, good subjective health, and regular social contacts, in turn, were determinants for a higher BAEL score. Further, the determinants were analysed separately by each cohort to illustrate the possible changes along time. In 1989 cohort, only SRH, loneliness and meeting friends were determinants for BAEL. In other cohorts, the findings remained essentially the same compared with the total sample (Table 3).

**Table 3:** Determinants for being actively engaged in life (BAEL score) categorised by cohorts combined (all) and separately (1989, 1999, 2009, 2019)

	Cohorts				
	All (*N* = 6054)	1989 (*n* = 552)	1999 (*n* = 2396)	2009 (*n* = 1492)	2019 (*n* = 1614)
	OR (95% CI)	OR (95% CI)	OR (95% CI)	OR (95% CI)	OR (95% CI)
Age group, years					
75	1.00 (ref)***	1.00 (ref)	1.00 (ref)***	1.00 (ref)***	1.00 (ref)***
80	0.82 (0.71–0.94)	0.94 (0.62–1.42)	0.81 (0.65–1.02)	0.82 (0.61–1.11)	0.67 (0.50–0.91)
85	0.52 (0.45–0.61)	0.71 (0.43–1.17)	0.51 (0.40–0.64)	0.46 (0.34–0.64)	0.44 (0.32–0.60)
90 and 95	0.43 (0.37–0.51)	NA	0.39 (0.30–0.52)	0.45 (0.32–0.62)	0.33 (0.24–0.46)
Sex, male	1.42 (1.26–1.59)***	0.99 (0.65–1.51)	1.55 (1.28–1.89)***	1.40 (1.11–1.77)**	1.35 (1.08–1.67)**
Widowed	0.72 (0.64–0.81)***	0.68 (0.46–1.01)	0.76 (0.64–0.91)**	0.75 (0.59–0.94)*	0.70 (0.56–0.88)**
Education < 8 years	0.75 (0.68–0.84)***	1.10 (0.73–1.66)	0.79 (0.67–0.94)**	0.90 (0.72–1.12)	0.74 (0.59–0.93)**
CCI^a^	1.01 (0.98–1.04)	1.02 (0.88–1.18)	0.99 (0.95–1.04)	1.01 (0.95–1.08)	1.02 (0.95–1.09)
Able to easily walk outdoors	1.42 (1.23–1.63)***	1.20 (0.75–1.94)	1.18 (0.95–1.47)	1.55 (1.18–2.05)**	1.73 (1.32–2.27)***
Needs daily help	0.89 (0.76–1.04)	1.26 (0.69–2.28)	0.84 (0.65–1.09)	0.88 (0.66–1.19)	0.88 (0.64–1.20)
Self-rated health good	2.12 (1.83–2.45)***	1.98 (1.24–3.18)**	1.95 (1.54–2.47)***	2.10 (1.58–2.79)***	2.26 (1.68–3.06)***
Suffering from loneliness constantly	0.24 (0.18–0.33)***	0.29 (0.09–0.92)*	0.28 (0.18–0.43)***	0.25 (0.13–0.51) ***	0.17 (0.09–0.31) ***
Meeting friends regularly	1.89 (1.69–2.12)***	2.18 (1.41–3.38)***	1.82 (1.52–2.19)***	2.18 (1.74–2.72) ***	1.82 (1.47–2.26) ***

*p < 0.05, **p < 0.01, ***p < 0.001 (Age groups’ p-values indicate linearity)

^a^Charlson Comorbidity Index [17]

**Fig. 1:**
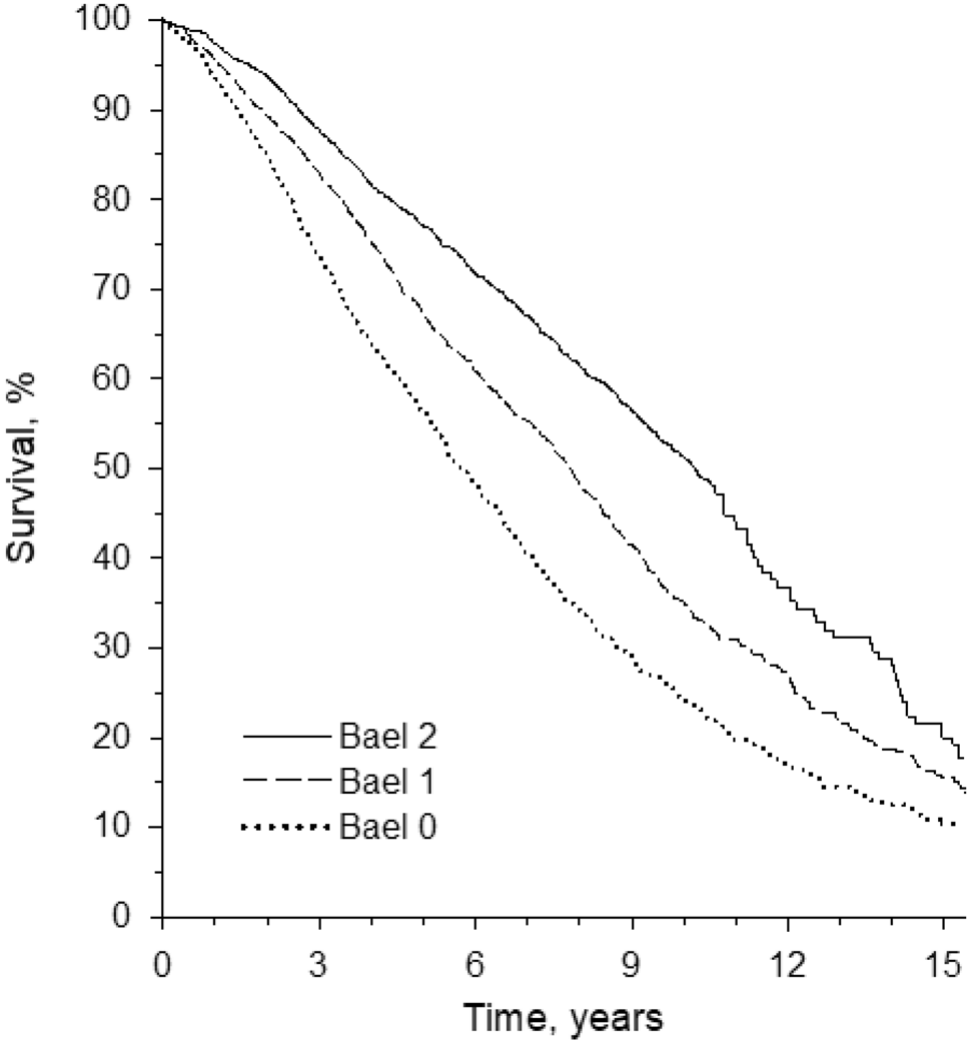
Kaplan–Meier survival curves for BAEL (being actively engaged in life) scores 0, 1, and 2

To explore how BAEL predicted mortality, we combined the 1989, 1999, and 2009 cohorts. Kaplan–Meier curves show how BAEL score predicted lower 15-year mortality (Fig. 1).

In Cox regression model and using BAEL score 0 as a reference, the HR for BAEL score 1 was 0.84 (95% CI 0.76–0.92) and for BAEL score 2 0.65 (95% CI 0.59–0.72) when adjusted for age, sex, and CCI. We repeated the analysis by adjusting for “able to easily walk outdoors” instead of “CCI”, and the results remained essentially the same (data not shown). Furthermore, the findings were similar when adjusted for “needs daily help” and dementia diagnosis (data not shown). A linear trend emerged from BAEL 0 to BAEL 2 in mortality (*p* < 0.001 for linearity).

## Discussion

In this study investigating four cohorts of older home-dwelling people, a significant increase occurred in the proportion of those being actively engaged in life over 3 decades, when measured as feeling needed and having plans for the future. Several characteristics, such as younger age, male sex, higher level of education, and good health, were associated with a higher BAEL score. The findings show that in addition to good perceived health and physical functioning, social contacts and not feeling lonely were determinants to being actively engaged in life. Finally, higher BAEL score predicted lower mortality, indicating that being actively engaged had a positive effect on survival.

The factors behind the higher level of engagement in life among urban home-dwelling Finnish older people in recent years are diverse. Participants in the more recent cohorts had a significantly higher education than those in the first cohorts, therefore, likely also reflecting a better financial status [18]. Our findings emphasise the importance of life-long socioeconomic factors for being actively engaged in life also in old age. People with a more advantaged socioeconomic background are generally considered to have better resources to take care of themselves and to promote a healthy lifestyle [19]. Besides socioeconomic factors, also biological factors may be involved. In a socioeconomically homogenous male cohort (the Helsinki Businessmen Study), cardiovascular risk at midlife predicted psychological well-being (including questions of BAEL) in old age, 29 years later [20].

As in previous studies [21–24], male sex, younger age, being married, and having a higher level of education were associated with higher odds for active ageing; in our study, these factors characterised those with higher BAEL scores. Compared with women, older men tend to be more engaged in socially oriented activities outside the home [25], which has been suggested to be one factor underlying better subjective well-being [26]. An increasing proportion of male participants were included in the more recent cohorts of our study [27], possibly being partly responsible for the greater proportion of higher BAEL scores in the later cohorts.

Declining mobility, increasing number of diseases, and increasing frailty with ageing might restrict participation in activities and active life, which has been observed also in a previous study [14]. However, it is interesting that in our stepwise logistic regression analysis, comorbidities or need for daily assistance were not significant determinants for BAEL. Our study emphasises the importance of meaningful social relationships as determinants for BAEL and active agency. Loneliness, meeting friends and good perceived health were determinants in all cohorts. The 1989 cohort was smaller and did not include 90- and 95-year-olds. Therefore, low statistical power may explain why some of the determinants did not reach significance. A Dutch study revealed that having plans and wishes was positively associated with older people’s life satisfaction [28]. Furthermore, when older people’s personal views on how to age successfully were queried, the majority valued engagement in activities and satisfying relationships over health issues [28]. It is obvious that ageing is not a static state, but instead a dynamic process reflecting an individual’s previous way of life and socioeconomic circumstances in earlier life-course [24].

Our results show that being actively engaged in life also protects against mortality. The mortality follow-up ranged up to 15 years, making the finding of prognostic value even more robust. This aspect has been seldom studied. However, there is some evidence that social engagement decreases the odds of mortality [29–31]. In a study investigating older African Americans, social engagement understood as purposeful activities and social interaction was also found to predict reduced mortality [31]. In the Helsinki Businessmen Study, BAEL predicted 12-year mortality (*p* < 0.001) among males with an average age of 82 years at baseline (unpublished observations).

Our study has some limitations. The population was an urban cohort, so the results may not be generalizable to older people living in other contexts or populations. Although the response rates are high, those with poorest cognition and major limitations in functioning did probably not respond. The concept of BAEL is still obscure, and we included only two questionnaire items on it. However, in our study, BAEL showed a significant relationship with demographics, health, functioning, social relationships, and mortality. BAEL showed predictive value for mortality even after adjustment for age, sex, and comorbidities.

Despite these limitations, the study also has notable strengths. The study population is representative of older home-dwelling people. The responders cover older people’s cohorts over 3 decades. The questionnaires were similar for all cohorts throughout the study. Although the response rates declined over time, the rates are high even in recent cohorts. The results contribute to the literature by strengthening previous findings and by presenting a simple way to assess active ageing with two subjective questions.

## Conclusions and implications

Being actively engaged in life is important in predicting survival beyond diseases or physical functioning. Becoming older and frail might cause withdrawal from social activities and participation due to decline in cognitive and physical competencies. Policies that promote various age-friendly solutions in the surrounding community should be supported to enable older people to take part in leisure and social activities, to maintain active agency, and to enhance their quality of life.

## Data Availability

The data concerning the current study are available from the corresponding author on reasonable request.

## References

[1] World Health Organization (2002) Active ageing: a policy framework. World Health Organization. https://apps.who.int/iris/handle/10665/67215. Accessed 20 Jan 2023

[2] Martin P, Kelly N, Kahana B (2015). Defining successful aging: a tangible or elusive concept?. Gerontologist.

[3] Bowling A, Dieppe P (2005). What is successful ageing and who should define it?. BMJ.

[4] Foster L, Walker A (2021). Active ageing across the life course: towards a comprehensive approach to prevention. Biomed Res Int.

[5] Phelan EA, Larson EB (2002). "Successful aging"–where next?. J Am Geriatr Soc.

[6] Phelan EA, Anderson LA, LaCroix AZ (2004). Older adults' views of "successful aging"–how do they compare with researchers' definitions?. J Am Geriatr Soc.

[7] Kusumastuti S, Derks MG, Tellier S (2016). Successful ageing: a study of the literature using citation network analysis. Maturitas.

[8] Urtamo A, Jyväkorpi SK, Strandberg TE (2019). Definitions of successful ageing: a brief review of a multidimensional concept. Acta Biomed.

[9] Boudiny K (2013). 'Active ageing': from empty rhetoric to effective policy tool. Ageing Soc.

[10] Douglas H, Georgiou A, Westbrook J (2017). Social participation as an indicator of successful aging: an overview of concepts and their associations with health. Aust Health Rev.

[11] Calderón-Larrañaga A, Hu X, Haaksma M (2021). Health trajectories after age 60: the role of individual behaviors and the social context. Aging (Albany NY).

[12] Rueda-Salazar S, Spijker J, Devolder D (2021). The contribution of social participation to differences in life expectancy and healthy years among the older population: A comparison between Chile, Costa Rica and Spain. PLoS ONE.

[13] Pitkala KH, Savikko N, Routasalo P, Derrickson H (2015). Group dynamics in older people’s closed groups. Findings from Finnish psychosocial group rehabilitation for lonely older people. Group therapy.

[14] Hladek MD, Zhu J, Buta BJ (2021). Self-efficacy proxy predicts frailty incidence over time in non-institutionalized older adults. J Am Geriatr Soc.

[15] Lee JW, Nersesian PV, Suen JJ (2022). Loneliness is associated with lower coping self-efficacy among older adults. J Appl Gerontol.

[16] Thanakwang K, Isaramalai SA, Hatthakit U (2014). Development and psychometric testing of the active aging scale for Thai adults. Clin Interv Aging.

[17] Charlson ME, Pompei P, Ales KL (1987). A new method of classifying prognostic comorbidity in longitudinal studies: development and validation. J Chronic Dis.

[18] Karppinen H, Pitkälä KH, Kautiainen H (2017). Changes in disability, self-rated health, comorbidities and psychological wellbeing in community-dwelling 75–95-year-old cohorts over two decades in Helsinki. Scand J Prim Health Care.

[19] Doménech-Abella J, Mundó J, Moneta MV (2018). The impact of socioeconomic status on the association between biomedical and psychosocial well-being and all-cause mortality in older Spanish adults. Soc Psychiatry Psychiatr Epidemiol.

[20] Strandberg TE, Strandberg AY, Pitkälä KH (2006). Cardiovascular risk in midlife and psychological well-being among older men. Arch Intern Med.

[21] Lu W, Pikhart H, Sacker A (2019). Domains and measurements of healthy aging in epidemiological studies: a review. Gerontologist.

[22] Lee WJ, Peng LN, Lin MH (2020). Determinants and indicators of successful ageing associated with mortality: a 4-year population-based study. Aging (Albany NY).

[23] Thoma MV, Kleineidam L, Forstmeier S (2020). Associations and correlates of general versus specific successful ageing components. Eur J Ageing.

[24] Rojo-Perez F, Rodriguez-Rodriguez V, Molina-Martinez MA (2022). Active ageing profiles among older adults in Spain: A Multivariate analysis based on SHARE study. PLoS ONE.

[25] Li YP, Lin SI, Chen CH (2011) Gender differences in the relationship of social activity and quality of life in community-dwelling Taiwanese elders. J Women Aging 23:305–320. 10.1080/08952841.2011.611052. (Erratum in: J Women Aging. 2012;24:94) 10.1080/08952841.2011.611052 22014220

[26] Dury S, Stas L, Switsers L (2021). Gender-related differences in the relationship between social and activity participation and health and subjective well-being in later life. Soc Sci Med.

[27] Öhman HR, Karppinen H, Lehti TE (2022). Secular trends in functional abilities, health and psychological well-being among community-dwelling 75- to 95-year-old cohorts over three decades in Helsinki. Finland Scand J Public Health.

[28] Huijg JM, van Delden AL, van der Ouderaa FJ (2017). Being active, engaged, and healthy: older persons' plans and wishes to age successfully. J Gerontol B Psychol Sci Soc Sci.

[29] Pitkala KH, Laakkonen ML, Strandberg TE (2004). Positive life orientation as a predictor of 10-year outcome in an aged population. J Clin Epidemiol.

[30] Tilvis RS, Laitala V, Routasalo P (2012). Positive life orientation predicts good survival prognosis in old age. Arch Gerontol Geriatr.

[31] Lamar M, James BD, Glover CM (2022). Social engagement and all-cause mortality: a focus on participants of the minority aging research study. Am J Prev Med.

